# Obstetric management of the most common autoimmune diseases: A narrative review

**DOI:** 10.3389/fgwh.2022.1031190

**Published:** 2022-11-23

**Authors:** Irene Fernández-Buhigas

**Affiliations:** ^1^ Obstetrics and Gynecology Department, Hospital Universitario de Torrejón, Madrid, Spain; ^2^ School of Medicine, Universidad Francisco de Vitoria, Pozuelo de Alarcón, Madrid, Spain

**Keywords:** pregnancy, autoimmune disease (AD), systemic lupus—erythematosus, antiphospholipid (Hughes) syndrome (APS), anti-Ro and anti-La antibodies, rheumatoid arthritis, sjogren's syndrome

## Abstract

Historically, women with an autoimmune disease (AD) could not get pregnant due to infertility frequently linked to the medical condition or because the pregnancy was contraindicated, as it could harm the mother and the future child. Sometimes, pregnancy was contraindicated because the medication needed to control the AD could not be given during pregnancy. All these items are no longer true nowadays. Fertility treatments have advanced, obstetric care is better, and the medical treatments of autoimmune diseases have progressed, so women with any kind of AD are encouraged to get pregnant, and their presence in obstetric clinics is arising. This is challenging for the obstetricians, as to be sure that these pregnancies are safe for the mother and the future child, the obstetricians need to know the natural evolution of these conditions, the impact of pregnancy and postpartum on the illness, and the impact of the AD in the pregnancy. In this narrative review, we aim to make a brief resume of the obstetric management of the most common diseases (Systemic lupus erythematosus, antiphospholipid syndrome, the Anti-Ro/SSA and Anti-La/SSB antigen-antibody systems, rheumatoid arthritis, Sjögren's syndrome and Undifferentiated systemic rheumatic disease and overlap syndromes).

## Introduction

Historically, women with an autoimmune disease (AD) couldn't get pregnant due to infertility frequently linked to the medical condition or because the pregnancy was contraindicated, as it could harm the mother and the future child. Sometimes, pregnancy was contraindicated because the medication needed to control the AD could not be given during pregnancy. All these items are no longer true nowadays. Fertility treatments have advanced, obstetric care is better, and the medical treatments of autoimmune diseases have progressed, so women with any kind of AD are encouraged to get pregnant, and their presence in obstetric clinics is arising. This is challenging for the obstetricians, as to be sure that these pregnancies are safe for the mother and the future child, the obstetricians need to know the natural evolution of these conditions, the impact of pregnancy and postpartum on the illness, and the impact of the AD in the pregnancy. An AD is characterized by a pathological response through autoantigens directed to normal body constituents. This response leads to an inflammatory cell injury or a functional disturbance with clinical manifestations (1, 2). AD can be classified as systemic (p.e. Systemic Lupus Erythematosus) or organ-specific (p.e. Sjogren's syndrome) and either chronic or acute. It usually involves T and B cell response, with the presence of specific antibodies.

Pregnancy and postpartum can modify the natural course of the AD, with exacerbations and recoveries. AD with a predominant cellular response, such as rheumatoid arthritis, generally tends to be less active during pregnancy. In contrast, those with a predominant humoral immunity, like Systemic Lupus Erythematosus, tend to flare up during pregnancy. Pregnancy changes can also impact the natural evolution of the AD. The increased intravascular volume can worsen the cardiac or renal function (worsening previous proteinuria), or the pregnancy-induced hypercoagulability can increase the thrombosis risk in these women. Typical pregnancy symptoms can mimic some active AD symptoms, and distinguishing between pregnancy complications such as preeclampsia and AD flare can be pretty challenging (3).

In general, the treatment of the AD aims to decrease the immune system (immunosuppressants) or block the inflammatory response that leads to tissue injury (anti-inflammatories) (4).

The immune response and the treatment used to treat the AD can interfere with the pregnancy evolution, so antibodies and medication should always be taken to account for counseling the pregnancy outcome and for following up correctly on the pregnancy and postpartum.

Periconceptional counseling is always essential, but in these women, it is vital. The pregnancy should be programmed when the AD is in low activity and with the appropriate medication to have a better pregnancy outcome. When pregnancy starts, these women should be seen at an early gestational age (before 9–10 weeks), as pregnancy loss is more common. If there has not been periconceptional counseling, medication should be adjusted to the pregnancy, and counselling about the pregnancy outcome should be given.

All these items lead to a need to care for each pregnant woman with an AD individually and with a multidisciplinary team (obstetrician, rheumatologist, etc.). In this narrative review, we aim to make a brief resume of the obstetric management of the most common diseases.

## Systemic lupus erythematosus (SLE)

It is a chronic autoimmune disease of unknown cause that can affect any body organ. The diagnosis is challenging, and several classifications have been published, but their usefulness in the clinic is limited (5–7). The diagnosis of SLE is generally based on clinical and laboratory findings after excluding alternative diagnoses. Serologic findings are important in suggesting the possibility of SLE, with some antibodies (p.e. anti-double-stranded deoxyribonucleic acid and anti-Smith) highly associated with this condition. Antinuclear antibodies (ANA) are positive in almost all SLE patients at some point in their disease, but their presence cannot always be associated with SLE. 15% of the United States population is ANA positive, but only around 10% of them have an AD (8). All these facts make SLE very heterogeneous, so individual counseling and multidisciplinary care are needed in all SLE patients.

### Preconceptionally counseling

Ideally, conception should be planned when the activity of the SLE is low or quiescent and when the medical condition is controlled with medication that is not harmful to the fetus (3, 9). So, pregnancy should not be encouraged until the activity is regulated for at least 6–12 months. The risk factors for maternal and fetal outcomes should be analyzed individually, and preventive strategies should be implemented. The European League Against Rheumatism (EULAR) recommendations (9) present a Checklist of parameters to be considered in preconceptionally counseling that includes SLE activity, obstetric history, and maternal characteristics.

An analytical evaluation should be performed at least 12 months before conception. It should include a renal function, complete blood count, liver function test, cardiac and hemostatic profile, and also an immunologic profile with anti-double-stranded DNA antibodies, Complement levels (C3, C4, CH50), antiphospholipid antibodies if previously negative (lupus anticoagulant, immunoglobulin G and M anticardiolipin antibodies, and IgG and IgM anti-beta2-glycoprotein I antibodies), and anti-Ro/SSA and anti-La/SSB if previously negative (10).

### Medication

All the medication taken by the women should be assessed, and if a teratogenic effect is detected should be changed. The safest immunosuppressors are Azathioprine (AZA) and 6-mercaptopurine; nevertheless, the lowest dose needed should be used (11). Sulfasalazine, although safe, can lead to a folate deficiency, so supplementation is needed (12). Cyclosporine is also safe, but the minimum dose should be used, and maternal blood pressure and renal function should be monitored (10). Tacrolimus is the more aggressive immunosuppressor used in SLE. Although it looks safe for the pregnancy, data is sparse, so obstetric precautions should be taken if needed (12). It is unclear whether Non-steroidal anti-inflammatory drugs (NSAIDs) could increase the risk of spontaneous abortion in women with difficulties in conceiving; therefore, it should be avoided. In the second and third trimesters, NSAIDs can cause oligohydramnios and ductal constriction, which should be monitored if they have to be used (13). Glucocorticoids are sometimes needed to control the disease if other medications are insufficient. When required, the lowest dose of prednisone should be used, and special care for monitoring fetal growth, maternal hypertension, and gestational diabetes should be taken (11, 14, 15).

There are some contraindicated drugs during pregnancy, and if pregnancy occurs during its use, the pregnancy outcome should be assessed. Cyclophosphamide should be avoided during the first 10 weeks of gestation, as it is associated with congenital malformations (11). Still, if used in the second or third trimester, although the data is sparse, it looks to have little or no impact on the fetus (16, 17). Mycophenolate mofetil, methotrexate, and leflunomide are contraindicated during pregnancy as they are associated with severe teratogenic effects (10).

Two medications are highly recommended in all SLE pregnant women. Those are:

- *Hydroxychloroquine*: is highly recommended before and during pregnancy as it reduces the risk of SLE flares and seems to lead to better pregnancy outcomes (18–20). We suggest continuing with the hydroxychloroquine during pregnancy if it was used before or starting with it preconceptionally or as soon as possible during pregnancy in all SLE pregnant women.
- *Low dose aspirin (LDA):* As shown in the ASPRE trial, 150mg per day of aspirin, started before 16 weeks, can reduce the risk of developing preterm preeclampsia in a high-risk population (21). SLE women are at high risk of developing it, around 14% higher risk than the general population (22). The use of a low dose of aspirin in all SLE pregnant women from 12 weeks until 36 weeks is recommended by the EULAR group (9), the US Protective Health Task Force (USPHTF) (23), and the American College of Obstetrics and Gynecology (ACOG) (24).

### Pregnancy follow-up

As the activity of the SLE is the primary indicator of the pregnancy prognosis, it is recommended to monitor the SLE activity by clinical history, examination, and laboratory tests (complete blood cell count, differential cell count, urinalysis results and proteinuria, anti-DNA, C3, and C4), at least once per trimester, and by the patient's clinical status (3).

Pregnancies should be followed up similarly to those with a high risk of hypertensive disorder and placental insufficiency (9). Routine ultrasonographic screening should be performed as usual for this population (first and second routine scans). In the second and third trimesters, special precautions should be taken to detect any fetal growth anomaly.

With this goal, we propose having the first appointment before ten weeks to evaluate all the preconception items and counsel the pregnancy outcome. Perform the first and second-trimester screening scans, and then start with surveillance of fetal growth and maternal clinical status every four weeks (9). This would be adjusted to any medical complication that could appear.

Guidelines are not clear about when to deliver these pregnancies. If no fetal event has occurred and maternal disease is well controlled, local guidelines for low-risk pregnancies could be followed. Still, induction between 39 and 40 weeks could also be recommended to reduce the risk of hypertensive disorder and placental insufficiency (9). So individual counseling should be performed, taking to account the individual risk of preeclampsia, fetal growth restriction, and perinatal and maternal clinical features.

### Special events during pregnancy

#### Exacerbation of SLE

The ranges of SLE flare during pregnancies vary from 7%–30% if low or quiescent activity of SLE was present six months before conception to 20%–60% if the activity was not controlled before (25–27). History of lupus nephritis, primigravida, and discontinuation of hydroxychloroquine, are also known factors of SLE exacerbation (10).

Signs of exacerbations may be a recent appearance of arthritis, arthralgias, edema, or fever of unknown origin. Also, the rise of anti-DNA antibodies or complement levels (complement levels are physiologically mildly elevated in pregnancy, so they must be carefully evaluated). If the clinical feature is mild, rest and paracetamol, along with the regular intake of hydroxychloroquine and topic steroids if cutaneous manifestation, should be enough. If the symptoms do not improve within two weeks, prednisone can be added. If the exacerbation is severe, high doses of corticosteroids could be needed, and close monitoring at the hospital should be done.

#### Lupus nephritis flare

The most complicated issue is differentiating lupus nephritis flare from preeclampsia, as it increases proteinuria, hypertension, thrombocytopenia, and worsening renal function. The onset of the overlapping symptoms before 20 weeks makes the lupus nephritis flare more common. Laboratory tests and clinical features could help us distinguish both entities (Table 1). The treatment consists of corticosteroids and immunosuppressors (AZA recommended). Suppose the severe flare does not respond to drugs with an acceptable safety profile. In that case, multidisciplinary care should be performed, and, occasionally, the end of the pregnancy and the use of embryotoxic drugs could be taken to a count (28).

**Table 1:** Lupus nephritis flare vs. preeclampsia.

	Lupus nephritis flare	Preeclampsia
Blood pressure	Normal or High	High
Onset	At any time in gestation	>20 weeks
Proteinuria	Present	Present
Urine Sediment	Active (red and white cells and cellular casts)	No active
Uric acid	Normal	High or rising
Anti-DNA antibodies	Rising	Stable or normal
Complement	Low	Normal
SLE nor renal symptoms	Could be present and rising	Stable

#### Postpartum

Women with active disease before conception and those with significant end-organ damage are at a greater risk of disease flare in the postpartum period compared with those with inactive disease, so it is recommended that one month after delivery, laboratory and clinical follow-up should be performed (10).

Breastfeeding is possible, and medication should be adjusted.

For future pregnancies, we must emphasize the importance of preconception preparation. Also, inform the women about the possible consequences of a new pregnancy if any event occurred during the pregnancy (Worsen of nephritis, flares, perinatal outcome …).

## Antiphospholipid syndrome (APS)

The APS is a systemic autoimmune illness characterized by venous or arterial thrombosis, obstetric complications, and the presence of antiphospholipid antibodies (aPL) (29), that is, Lupus anticoagulant (LA), anticardiolipin antibody IgM and/or IgG (aCL) and Anti-b2 glycoprotein-I antibody (aB2GP1) IgG and or IgM. APS can be associated with other AD, especially in SLE. Around 30% of women with SLE have a clinically significant aPL profile (30), and, from the women with APS, about 36% associate SLE and around 5% a Lupus-like syndrome (31).

To make the APS diagnosis, we have to follow the updated Sapporo criteria (32) (Table 2). It should meet at least one clinical criterion and one laboratory criterion to confirm the syndrome. Classification of APS should be avoided if the aPL are detected in less than two testings and at least 12 weeks apart or if the clinical manifestation and the aPL detection are separated for more than five years.

**Table 2:** Updated Sapporo criteria for the diagnosis of APS (at least one clinical criterion and one laboratory criteria).

Clinical criteria	Laboratory criteria
*Vascular thrombosis (1 or more)*^a^*:* Arterial thrombosis Venous thrombosis Small vessel thrombosis	Lupus anticoagulant (LA) present in plasma, on 2 or more occasions at least 12 weeks apart. Anticardiolipin (aCL) antibody of IgG and/or IgM in serum or plasma, present in medium or high titer on 2 or more occasions at least 12 weeks apart. Anti-b2 glycoprotein-I antibody of IgG and/or IgM isotype in serum or plasma in titer >99th centile, present in 2 or more occasions at least 12 weeks apart Investigators are strongly advised to classify APS patients in studies into one of the following categories: I: more than one laboratory criteria present (any combination) IIa: LA present alone IIb: aCL antibody present alone IIc: anti-b2 glycoprotein-I antibody present alone.
*Obstetric complications:* 1 or more unexplained deaths of a morphological normal fetus at or beyond 10 weeks gestation 1 or more premature births of morphological normal fetus (<34 weeks) because of: Eclampsia or sever preeclampsia Placental insufficiency^b^ 3 or more unexplained consecutive spontaneous abortions before 10 weeks, with maternal anatomic or hormonal abnormalities and paternal and maternal chromosomal causes excluded.	

^a^ A thrombotic episode in the past could be considered a clinical criterion if thrombosis is proved by appropriate diagnostic means and that no alternative diagnosis or cause of thrombosis is found. Superficial venous thrombosis is not included in the clinical criteria.

^b^ Placental insufficiency includes (i) abnormal or non-reassuring fetal surveillance test(s), (ii) abnormal Doppler flow velocimetry waveform analysis suggestive of fetal hypoxemia, (iii) oligohydramnios, (iv) a postnatal birth weight less than the 10th percentile for the gestational age. Adapted from Miyakis et al. (32).

Some women do not meet the Sapporo criteria but have an aPL profile. It is controversial if these women are at high risk of pregnancy complications, infertility, or thrombosis. Still, most of the literature suggests that these women are not an at-risk group (33–39).

APS can be further classified according to the clinical condition that confirms the syndrome in (40)

- *Thrombotic APS*: when one or more vascular thrombosis occurs (Arterial thrombosis, venous thrombosis, or small vessel thrombosis)
- *Obstetric APS:* when an obstetric complication occurs, that is, one or more unexplained deaths of a morphologically normal fetus at or beyond ten weeks gestation; one or more premature births of morphologically normal fetus/es (<34 weeks) because of eclampsia or severe preeclampsia, or placental insufficiency (abnormal or non-reassuring fetal surveillance test, abnormal Doppler flow velocimetry waveform analysis suggestive of fetal hypoxemia, oligohydramnios, or postnatal birth weight less than the 10th percentile for the gestational age); or three or more unexplained consecutive spontaneous abortions before ten weeks, with maternal anatomic or hormonal abnormalities and paternal and maternal chromosomal causes excluded.
- *Catastrophic APS*: when the syndrome is life-threatening and characterized by thrombotic complications affecting multiple organs simultaneously or over a short period.

High levels of LA in women with APS have been correlated with worse obstetric outcomes (41, 42), and the triple positivity (LA, aCL, and aB2GP1 positive test) also appears to be associated with worse outcomes (43–46).

### Obstetric management

The ultrasound follow-up should be like the one performed in SLE. The best timing for delivery should be discussed with the patient. Guidelines are not clear about the best moment to finish the pregnancy, but considering the personal obstetric history and the risk of adverse perinatal outcomes, delivery between 39 and 40 weeks could be recommended.

#### Apl positive with no APS criteria

Although these women are not considered to be at a high risk of pregnancy loss, although controversial, it is generally considered to be a risk factor for preeclampsia (3, 37, 46–48). With very low-quality scientific evidence, the use of low-dose of aspirin (LDA) could be considered to reduce their risk (3, 9) [150 mg per day until 36 weeks of gestation (21)] if the individual circumstances could benefit (e.g., Triple positive aPL, strong positive LA, IVF, etc.)

#### Thrombotic APS

Women with prior thrombosis are generally treated with oral anticoagulants and sometimes with a low dose of aspirin (LDA). During pregnancy, the oral anticoagulant should be changed to low molecular weight heparin (LMWH) in the therapeutic dose (3, 9). In postpartum, the previous oral anticoagulant can be introduced if compatible with breastfeeding. LDA should continue with 150 mg daily (risk-benefit should be considered for continuing or not after 36 weeks).

#### Obstetric APS

Pregnancy is a risk factor for thrombosis, so in these women, prophylactic LMWH should be started when pregnancy begins and continued until 6–12 weeks postpartum. As the risk of preeclampsia is also higher in this group, LDA should also be started at 150 mg per day until 36 weeks of gestation (3, 9, 21).

Sometimes despite the correct treatment of the Obstetric APS, a poor outcome appears. This is known as refractory Obstetric APS. Some alternative therapies have been tried, such as hydroxychloroquine, intravenous immunoglobulin, or prednisone, with promising results, but nowadays, we cannot strongly recommend them as the evidence is scarce (49–51).

#### Postpartum

During postpartum, the antithrombotic medication should be changed to oral anticoagulant at the thrombotic APS, as they are safe during breastfeeding. In cases of Obstetric APS, prophylactic LMWH should be continued until 6–12 weeks postpartum.

For future pregnancies, we must emphasize the importance of preconception preparation.

## The anti-Ro/SSA and anti-La/SSB antigen-antibody systems

These antibodies can be present in different AD such as SLE (30%–50%), Sjögren syndrome (60%–90%), Rheumatoid arthritis (11%), etc (52). The presence of anti-Ro/SSA and/or anti-LA/SSB associates a high risk of several fetal and infant manifestations. This could be transient and spontaneously resolved (10% of neonatal lupus erythematosus, 20% transient thrombocytopenia, 30% mild transient transaminitis) or as severe as complete (third-degree) fetal heart block (2% on pregnancies with no prior infant affected with cutaneous or cardiac heart block, and 13%–18% if there was a previous infant affected) (3). Approximately 20% of children with complete cardiac heart block die *in utero* or within the first year of life, and more than 50% will need a pacemaker (53). In most cases, congenital heart block appears between 18 and 24 weeks and rarely after 26 weeks.

Most guidelines recommend *fetal echocardiogram monitoring* once a week from 16 to 26 weeks and less frequently afterward in pregnancies with a prior infant affected. But it is controversial if this should be done in those women with no previous pregnancy affected, as the global risk is low. Nevertheless, as intensive surveillance is well accepted and carries no risk, it is also recommended for these women (3, 9, 52, 54).

There is no proven treatment for complete fetal heart block (55–58). Third-degree heart block is always irreversible despite the therapy used, second-degree heart block will also progress despite the therapy used (59), and the studies with first-degree heart block are inconsistent (60, 61).

Recent data suggest the potential benefit of *hydroxychloroquine* in preventing the recurrence of complete fetal heart if there was a previous infant affected (62, 63) or if anti-Ro/SSA and/or anti-LA/SSB antibodies were present (62, 64). That is why most guidelines recommend using hydroxychloroquine in all pregnancies with anti-Ro/SSA and/or anti-La/SSB (3, 9, 52).

The use of *dexamethasone* to treat fetal heart block is controversial. There has not been a proven beneficial effect in preventing fetal heart block (65). In some studies, it appears that dexamethasone can reverse carditis and incomplete fetal heart blocks (55, 57, 66–68), which is why some guidelines recommend the use of dexamethasone in first and second-degree fetal heart block after assessing the risk and benefits (3, 52). Nevertheless, a systematic review in 2019 did not find any improvement in the use of antenatal corticosteroids alone or in combination in fetal or neonatal morbidity or mortality if fetal second-degree heart block had occurred (69).

*Intravenous immunoglobulins* and *plasmapheresis* in some small studies have been shown to have potential roles in preventing the recurrence of fetal heart block (64, 70–72), and in combination with corticosteroids seem to revert second-degree heart block (73, 74). Still, further and more extensive research should be carried out.

The delivery timing will be in accordance with the fetal status. Suppose there has not been any fetal manifestation, and the concomitant disease does not recommend any particular delivery timing. In that case, there is no evidence that a rapid induction would benefit the mother or the fetus, so local guidelines for low-risk pregnancies should be followed.

### Postnatal counseling

In these women is essential to counsel about the subsequent pregnancies. If the newborn had no manifestations, the risk of having a child with a heart block is about 2%. But if the newborn had a heart block, the risk of recurrence is six to ten times higher, and if the manifestation was neonatal cutaneous lupus, the risk of having a baby with a heart block is 13%–18% (62). It will be necessary then to counsel about using preventive hydroxychloroquine in future pregnancies.

## Rheumatoid arthritis (RA)

Rheumatoid arthritis (RA) is a chronic inflammatory disease characterized by joint swelling, joint tenderness, and destruction of synovial joints, leading to severe disability and premature mortality, and usually accompanied by autoantibodies that can precede the clinical manifestations for many years (75). The diagnosis is challenging. The 1987 American College of Rheumatology (ACR; formerly the American Rheumatism Association) criteria (76) focuses on distinguishing patients with RA and those with another inflammatory synovitis. The 2010 American College of Rheumatology (ACR)/European Alliance of Associations for Rheumatology (EULAR) classification criteria (75) tries to identify patients in earlier stages, where treatment could prevent patients from reaching the chronic erosive disease state. Using this new classification, younger women are diagnosed and treated, and their presence in the obstetric clinic is also arising.

Commonly, RA activity improves during gestation (around 60%) and flares during the several months postpartum in about 46.7% (77). But in half of them, flares can happen, or the activity can remain during pregnancy (78), so we have to be careful with the treatment used.

### Preconceptionally counseling

Ideally, counseling about treatment and changing those with teratogenic effects should be done before pregnancy. If not, it should be done as early as possible, and counseling about teratogenic treatment should be done.

In well-controlled RA, the pregnancy outcomes are not worsened. Still, the overall risk of adverse perinatal outcomes is augmented (hypertensive disorders, intrauterine growth restriction, preterm delivery, and cesarean delivery) (79, 80). So ideally, RA should be controlled 6–12 months before the pregnancy with no teratogenic medication (p.e. sulfasalazine, hydroxychloroquine, tumor necrosis factor alfa inhibitor).

As it is associated with Anti-Ro/SSA and/or anti-LA/SSB in around 11%of cases (52), we should test for these antibodies before pregnancy or as soon as possible during pregnancy if not previously performed (3).

### Pregnancy and postpartum follow-up

If the RA is well controlled and is not associated with anti-Ro/SSA or anti-LA/SSB antibodies, they can follow standard pregnancy care, and in each appointment, we should question possible flares. In the case of flares or if the RA was not controlled, close monitoring of fetal growth and hypertensive disorders should be performed (3).

Delivery timing should be in accordance with fetal and maternal well-being. If both manage pregnancy well, local guidelines for low-risk pregnancies should be followed. If maternal symptoms are limiting, the best time for delivery should be discussed with the patient, trying to achieve, if possible, 39 weeks of gestation.

In postpartum, as flares are more common, close monitoring should be performed because caring for a newborn may be very difficult with an RA flare. This fact should be advised to the women, so prompt treatment can be started as soon as the flare appears. Most medications are compatible with breastfeeding and should be used if necessary (3).

## Sjögren's syndrome (SS)

It is a chronic, multisystemic AD characterized by lymphocytic infiltration of the lacrimal and salivary glands provoking the secretory function loss of these glands and infiltration that may also occur in other organs. Symptoms range from xerophthalmia, xerostomia, fatigue, myalgia, and arthralgia to severe systemic symptoms with cutaneous, vascular, renal, pulmonary, or neurological involvement (81). It can be present alone as primary Sjögren syndrome (pSS) or associated with an underlying AD (commonly RA or SLE). Affected women with SS are likely to have worse perinatal outcomes than the general population, but data on pSS are scarce, as SS is usually associated with Anti-Ro/SSA. The Brazilian Society of rheumatology has recently published some recommendations on the obstetric care of these pregnancies (52). The British Society for rheumatology has also published a guideline for managing adults with pSS (54). Both conclude that pregnant women with pSS are at high risk of adverse perinatal outcomes [premature birth, lower neonatal average weight, intrauterine growth restriction (82)], and special care should be taken. They recommend that the disease should be controlled for at least six months before pregnancy and that the profile of aPL and anti-Ro/SSA antibodies should be updated. The British guideline (54) recommends using LDA to improve placental implantation, but with a very low level of scientific evidence.

We recommend establishing if SS is associated with SLE, AR, or anti-Ro/SSA. If, so, the obstetric management should be as explained before in each AD. If there are no other associations, we recommend close monitoring of the pregnancy by ultrasound (monthly), but no further treatments should be needed. If no fetal or maternal events have occurred, the delivery timing is unclear, and local guidelines for low-risk pregnancies should be followed.

SS is likely to worsen during pregnancy and postpartum, mainly due to a worsening of pulmonary hypertension (83). Women should be aware of this, and close monitoring of clinical symptoms should be performed to promptly identify and treat if necessary.

## Undifferentiated systemic rheumatic disease and overlap syndromes

Some patients do not entirely fit all diagnostic criteria for any rheumatoid disease, although they have systemic syndromes, and they will be undiagnosed during 5–10 years (84, 85). The follow-up of these women is more complicated, as it should be according to their primary rheumatological symptoms but taking to account every antibody or symptom that could interfere with the pregnancy (for example, anti-Ro/SSA or Anti-La/SSB antibodies). As in all the other medical conditions, but more importantly in these women, as the syndrome could be more complex, a multidisciplinary follow-up should be done.

## Conclusion

Pregnant women with an AD should be followed up by a multidisciplinary team (obstetricians, rheumatologists, etc.).
